# Embodied pain in fibromyalgia: Disturbed somatorepresentations and increased plasticity of the body schema

**DOI:** 10.1371/journal.pone.0194534

**Published:** 2018-04-06

**Authors:** Endika Martínez, Zigor Aira, Itsaso Buesa, Ibane Aizpurua, Diego Rada, Jon Jatsu Azkue

**Affiliations:** 1 Department of Neurosciences, School of Medicine and Nursery, University of the Basque Country, UPV/EHU Barrio Sarriena s/n, Leioa, Bizkaia, Spain; 2 Preventive Medicine and Public Health Department, School of Pharmacy University of the Basque Country, UPV/EHU Paseo de la Universidad, Vitoria-Gasteiz, Araba, Spain; University of Exeter, UNITED KINGDOM

## Abstract

Fibromyalgia syndrome (FMS) is a highly prevalent, chronic musculoskeletal condition characterized by widespread pain and evoked pain at tender points. This study evaluated various aspects of body awareness in a sample of 14 women with FMS and 13 healthy controls, such as plasticity of the body schema, body esteem, and interoceptive awareness. To this end, the Rubber Hand Illusion (RHI), the Body Esteem Scale (BES), and the Body Perception Questionnaire (BPQ) were used, respectively. Consistent with increased plasticity of the body schema, FMS patients scored higher, with large or very large effect sizes, across all three domains evaluated in the RHI paradigm, namely proprioceptive drift and perceived ownership and motor control over the rubber hand. Scores on all items addressed by the BES were consistently lower among FMS subjects (2.52, SEM .19 *vs* 3.89, SEM .16, respectively, p < .01, Cohen’s *d* = .38-.66). In the FMS sample, BES scores assigned to most painful regions also were lower than those assigned to the remaining body sites (1.58, SEM .19 *vs* 2.87, SEM .18, respectively, p < .01). Significantly higher scores (p < .01, Cohen’s *d =* .51-.87) were found in the FMS sample across awareness (3.57 SEM .15 *vs* 1.87 SEM .11), stress response (3.76 SEM .11 *vs* 1.78 SEM .11), autonomic nervous system reactivity (2.59 SEM .17 *vs* 1.35 SEM .07), and stress style 2 (2.73 SEM .27 *vs* 1.13 SEM .04) subscales of the BPQ. Intensity of ongoing clinical pain was found to be strongly correlated with interoceptive awareness (r = .75, p = .002). The results suggest a disturbed embodiment in FMS, characterized by instability of the body schema, negatively biased cognitions regarding one’s own body, and increased vigilance to internal bodily cues. These manifestations may be interpreted as related with the inability of incoming sensory inputs to adequately update negatively biased off-line somatorepresentations stored as long-term memory.

## Introduction

Fibromyalgia syndrome (FMS) is a chronic musculoskeletal condition characterized by widespread pain and evoked pain at tender points [1–2]. Most patients with FMS also present with co-morbid anxiety and depression, and common findings also include fatigue and non-restorative sleep, memory and cognitive impairment, muscle stiffness, and gastrointestinal disorders. In addition, findings from several studies are consistent with central sensitization in FMS patients [3–5]. Up to 5.8% of the population of industrial countries may suffer from FMS [6–9].

The experience of pain is modulated on a cognitive level depending on attention, anticipation, emotion and memory of previous pain [10–12]. In addition, the ability to localize and confine a sensation to the body requires an intact body representation, and there is increasing evidence supporting the association of chronic pain with disturbed mental representations of the body [13–15]. Two concepts commonly referred to in the context of body awareness are body schema and body image, consistent with the notion that perception and action require different sensory signal processing as posed by the general functional hypothesis. Body schema is viewed as the dynamic, action-oriented implicit representation of one’s own body, which reflects the position and movement of the body in space, whereas body image refers to a cognitive and interpretative representation that integrates the conscious perceptual corporeal experiences and contributes to beliefs and attitudes towards the body [16–18]. The pain experience is an embodied one, inasmuch as the body is one’s way of experiencing the world and harbors the body schema which makes a person’s pain experience possible. Maintenance of the body schema and body image depends on multisensory bodily inputs, and there is growing evidence that both body schema and body image may be altered in patients suffering from chronic pain. In patients presenting with complex regional pain syndrome (CRPS), for example, mental representation of movement of the painful body part has been found to be slower [19–20], and visuospatial perception and laterality recognition may be impaired [21–22]. Patients with this condition also often present with altered motor performance, including limited range of motion, weakness or dystonia [23–26]. In addition, a number of studies in patients suffering from phantom pain or CRPS have reported distorted perceptions of the painful body parts [27–31]. In patients with FMS, poor balance and higher frequency of falls may be seen as indications of disturbed sensorimotor function and an altered body schema [32–33]. In addition, FMS patients perceive enlarged body size and shrinkage of the surrounding space during exacerbations of pain [34], suggesting a positive link between distortion of the body image and pain. Such relationship has been confirmed by direct correlations between poor body image and severity of the clinical status and ongoing pain [35].

On the other hand, interoception refers to the sense of the physiological condition of the body [36]. Interoceptive accuracy has been found to be positively linked with anxiety [37], while diminished interoception may be associated with depression and alexithymia [38–40]. Despite the fact that FMS is comorbid with anxiety and depression, the involvement of alterations of interoceptive awareness in this condition has not yet been established.

In order to gain insight into embodied pain in FMS, the current study explored various fundamental aspects of body awareness such as plasticity of the body schema, body image and interoceptive awareness. To this end, we administered the rubber hand illusion paradigm both to a sample of women with FMS and a group of healthy controls, and we used standard, self-administered questionnaires such as the Body Esteem Scale (BES) and the Body Perception Questionnaire (BPQ) to assess body image and interoceptive awareness, respectively.

## Methods

### Study participants and ethical statement

A convenience sample of fourteen right-handed women with a formal diagnosis of fibromyalgia syndrome was evaluated (average age 54.35 years, SEM 1.89), as well as 13 age-matched, healthy right-handed women which served as controls (average age 53.86 years, SEM 3.30). Patients were recruited February 2015 from a local support group through advertisements and informative presentations. No one participant had previously been subjected to the rubber had illusion (RHI, cf. below). The study protocol was approved by the Ethical Review Board of the University of the Basque Country and all participants provided written informed consent before participating.

### Pain and clinical status

Pain, clinical status and body perception were assessed by using self-administered instruments. Subjects provided an overall measure of pain severity on a visual analog scale (VAS), and the Spanish version of the short form of the Brief Pain Inventory (BPI-SF) [41] was used to assess the impact of ongoing pain on daily function. The Spanish version of the Fibromyalgia Impact Questionnaire (FIQ) [42–43] was used to assess physical functioning, work status, depression, anxiety, sleep, pain, stiffness, and fatigue. Health-related quality of life was evaluated by using the SF-12 Health Survey, a multipurpose survey comprised by 12 questions selected from the SF-36 Health Survey [44] that provides scores in both mental and physical domains as Mental Component Summary and Physical Component Summary scales, respectively.

### Rubber hand illusion

The RHI [5] is a well known somatosensory paradigm that can be induced when the experimental subject views a life-sized rubber hand being stroked by a paintbrush while his/her hand is hidden out of sight yet likewise stroked by another paintbrush. Simultaneous stroking of both the real and the rubber hand creates a sensory conflict between what one sees and feels, and the paradigm evaluates how the brain resolves such conflict by adapting the underlying body representation to embody the rubber hand. The RHI is thought to be based on visuotactile integration and can be used for assessing self-attribution and plasticity of the body schema [45–49]. Usual behavioral measures of the RHI include a drift of the perceived position of the subject’s hand toward the rubber hand, which is known as *proprioceptive drift*, as well as perceived appropriation (*ownership*) and motor control (*agency*) over the rubber hand.

**Fig 1:**
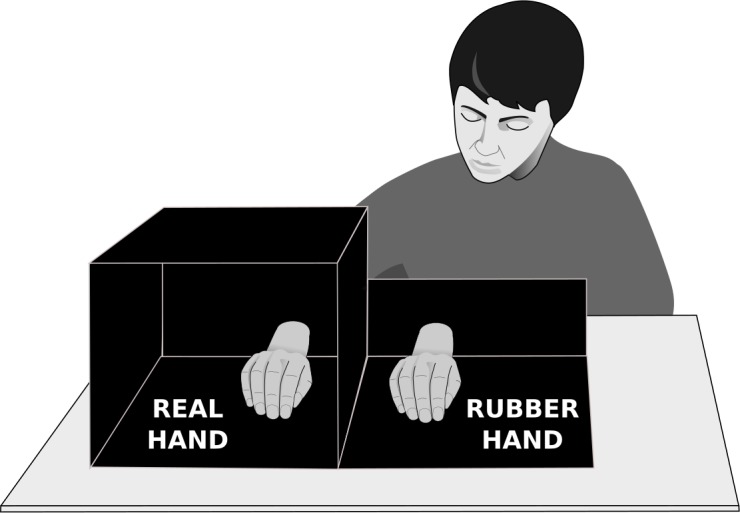
Experimental setting for inducing the rubber hand illusion. The subject was sitting at a table with eyes fixed on the rubber hand and her actual right hand placed inside an opaque box. The positions of the chair and the box both were adjusted so that the subject had the fake hand, but not the real one in sight. Small paint-brushes were used to synchronously stroke both the rubber hand and the subject’s hidden hand.

In the present study, each participant sat at an office table and placed her right hand inside an opaque plexiglas box (Fig 1). A rubber hand had been placed palm down between the real hand and the body mid-line. The subject had direct visual access to the fake hand, whereas her real hand was kept hidden inside the box. Two round paint-brushes (diameter about 1 cm) [50] were used for brushing both the patient’s right hand and the rubber hand in a synchronous manner for 1 min, starting at the middle phalanx on each finger and ending at the fingernail. A questionnaire based on items from previously published studies [51–58] was administered immediately thereafter to assess the perceived location of the real and rubber hands (5 items), as well as perceived appropriation (*ownership*) and motor control (*agency*) over the rubber hand (7 and 6 items, respectively). Responses were measured on 5-point Likert scales (1—*total disagreement*, 5—*total agreement*).

### Body esteem and interoceptive awareness

Body esteem, an important dimension of self-esteem, was measured by using the Body Esteem Scale (BES) [59]. This instrument evaluates perception and self-evaluation of one’s body by measuring the feeling towards various body parts and functions on a 5-point Likert scale (1 labeled as *very negative* and 5 as *very positive*).

The Body Perception Questionnaire (BPQ) [60] was used as a measure of self-rated bodily awareness. This tool uses 5-point scoring scales (1 denoting no awareness at all, whereas 5 indicates permanent awareness) to measure body perception and interoceptive awareness on four subscales, including awareness (perception of bodily processes, e.g. swallowing), stress response (perception of bodily changes in stressful situations), autonomic nervous system reactivity (perception of one’s own autonomic nervous system reactions, e.g. beating of one's heart), and stress style (e.g. frustration or dizziness).

### Data analysis and statistics

The SPSS® v. 22 statistical package (SPSS, Chicago, IL) was used for all statistical analyses. Normality of distribution was checked using the Shapiro-Wilk test, and the unpaired-sample two-tailed *t*-test or the Mann-Whitney-Wilcoxon *U*-test were used to investigate statistical significance of means differences between the two study groups.

Alpha values of .05 and .01 were used as criteria of statistical significance (indicated where appropriate). Primary data are presented as means and standard errors, and effects sizes are presented as Cohen’s *d* (95% confidence intervals). Likert scale data from individual questionnaire items were treated as ordinal data, whereas subscale scores were treated as interval data.

## Results

### Baseline clinical status of FMS patients

Subjects in the FMS sample scored an average of 88.38 (SEM 2.72) on the FIQ (data summarized in Table 1), which was slightly above the reported values of ca. 76 by studies in this geographic area [61–62]. Ongoing pain intensity was 8.89 (SEM .98) on the VAS, and scores on the Brief Pain Inventory averaged 7.39 (SEM .37), which can be considered as indicative of severe clinical pain with high interference with daily function. Differences on both pain measures relative to healthy controls were statistically significant. In addition, scores on the Physical- and Mental Component Summaries of the 12-Item Short-Form Health Survey (SF-12) both were significantly lower, i.e. they reflected an inferior clinical status, in the FMS sample (33.27, SEM 1.98 in the FMS sample, vs 23.10, SEM .82 in healthy controls).

**Table 1:** Ongoing pain and clinical status in the two study groups.

	FMS	Healthy Controls
Fibromyalgia Impact Questionnaire (FIQ)	88.38 (2.72)	–
Brief Pain Inventory–Short Form	7.39 (.37) **	.16 (.09)
Pain intensity (Visual Analog Scale)	8.89 (.28) **	.15 (.12)
SF12 –Physical Component Summary	23.10 (.82) **	53.68 (1.23)
SF12 –Mental Component Summary	33.27 (1.98) **	54.08 (2.09)

Subjects in the FMS sample had lower health indicators in terms of physical and mental summary scales of the 12-Item Short-Form Health Survey (SF-12), and exhibited significantly higher levels of clinical pain intensity on the VAS. The Fibromyalgia Impact Questionnaire (FIQ) was administered only to FMS subjects. Data are presented as mean (SEM).

** p < .01 on the Student’s *t* test for independent samples.

### Rubber hand illusion

FMS patients scored higher across all items of the assessment questionnaire (Table 2; raw data provided in S1 File). Observed effect sizes were large or very large across the vast majority of the items, with the sole exception of item 3 within the *ownership* domain (Cohen’s *d* < .5). Albeit high, effect sizes in the *ownership* domain were generally lower than in *proprioceptive drift* and *agency* domains, and differences between the two groups failed to attain statistical significance. Differences in scorings between groups were largest in the *proprioceptive drift* domain, in which statistically significant differences (Mann-Whitney-Wilcoxon *U*-test) and large effect sizes were found across all presented items (e.g. Cohen’s *d* value of 3.10 on the statement *It seemed as if the rubber hand and my own hand were approaching*).

**Table 2:** Assessment of the RHI in fibromyalgia and healthy controls.

	FMS	Healthy controls	Effect size
**Proprioceptive drift**			
It seemed like the rubber hand was where my own hand was	4.07 (.22) *	3.08 (.38)	.88 (.07–1.68)
I felt as if my own hand was drifting towards the rubber hand	3.14 (.25) **	1.38 (.24)	1.95 (1.01–2.88)
It seemed as if the touch I was feeling came from somewhere between my own hand and the rubber hand	3.07 (.32) **	1.38 (.24)	1.61 (.73–2.50)
It seemed (visually) as if the rubber hand was drifting towards my own hand	3.21 (.28) **	1.23 (.12)	2.46 (1.44–3.48)
It seemed as if the rubber hand and my own hand were approaching	3.50 (.25) **	1.23 (.21)	3.10 (1.96–4.24)
**Ownership**			
It seemed like the rubber hand belonged to me	3.92 (.26)	3.15 (.37)	.60 (-.18–1.38)
It seemed like the rubber hand began to resemble my real hand	3.50 (.32)	2.76 (.37)	.57 (-.21–1.35)
It seemed like I was looking directly at my own hand, rather than at a rubber hand	4.07 (.28)	3.46 (.41)	.46 (-.31–1.24)
It seemed like the rubber hand was part of my body	4.07 (.26)	3.15 (.37)	.78 (-.01–1.58)
It seemed like the rubber hand was my hand	3.85 (.27)	3.15 (.38)	.57 (-.21–1.35)
The rubber hand began to resemble my own hand in terms of shape, skin tone, freckles or some other visual features	3.50 (.27)	2.53 (.44)	.72 (-.07–1.51)
It seemed as if I was feeling the touch of the paintbrush in the location where I saw the rubber hand touched	4.21 (.21)	3.15 (.50)	.75 (-.03–1.55)
**Agency**			
It seemed like I was unable to move my own hand	3.92 (.24) **	1.53 (.31)	2.34 (1.34–3.34)
It seemed like I could not really tell where my hand was	3.50 (.22) **	1.69 (.32)	1.76 (.85–2.66)
It seemed like my own hand had disappeared	3.78 (.26) **	2.00 (.39)	1.47 (.60–2.33)
It seemed like my own hand was out of my control	3.57 (.22) **	1.54 (.27)	2.24 (1.26–3.23)
It seemed like I could move the rubber hand if I would like	3.21 (.33)	2.38 (.43)	.58 (-.19–1.37)
It seemed like I was in control of the rubber hand	3.28 (.33)	2.38 (.43)	.63 (-.15–1.42)

FMS patients scored generally higher relative to healthy controls across all items in all three domains of the questionnaire. Data from 5-point Likert scales (1 –totally disagree, 5- totally agree) are presented as means (SEM; * p < .05, ** p < .01 on the Mann-Whitney-Wilcoxon *U*-test). Effect size is provided as Cohen’s *d* (95% CI).

### Lower body esteem in FMS

The Body Esteem Scale (BES) was used here to evaluate body satisfaction. We found that the average global BES score was lower among FMS subjects (2.52, SEM .19) relative to healthy controls (3.89, SEM .16), the difference being statistically significant (p < .01 on the Student’s *t*-test for independent samples), i.e. patients reported less satisfaction with their bodies than did healthy individuals. Scores assigned by subjects from the FMS sample were consistently lower across all items within the BES, effect sizes being moderate to high with Cohen’s *d* values ranging between .38 and .66 (Table 3). Inter-group comparisons revealed statistically significant differences in BES scores on items addressing 14 out of 18 body regions across all four main body segments evaluated (head, superior and inferior limbs, and trunk). We also wished to ascertain whether body regions receiving lower scores corresponded to those primarily affected by ongoing clinical pain. To this end, we extracted the BES score assigned by each FMS patient to the body site regarded as most painful, and we then obtained the average of scores assigned to the remaining body regions addressed by the BES, which were either less painful or not painful at all. We found that average BES scores corresponding to most painful regions in the FMS sample were indeed lower (1.58, SEM .19) than those assigned to less painful or non-painful sites (2.87, SEM .18). Differences were statistically significant (p < .01 on the Student’s *t-*test for independent samples).

**Table 3:** Scores from FMS patients and healthy controls on the Body Esteem Scale.

	FMS	Healthy Controls	Effect size
Body scent	3.21 (.23)	3.69 (.39)	.38 (-1.18-.3)
Appetite	3.64 (.17) *	4.38 (.28)	.39 (-.04–1.54)
Nose	3.42 (.29)	3.61 (.24)	.38 (-.58-.96)
Physical stamina	1.92 (.30) **	3.92 (.30)	.45 (.85–2.67)
Reflexes	2.85 (.39) **	4.50 (.15)	.44 (.60–2.38)
Lips	3.43 (.31) *	4.31 (.21)	.40 (.09–1.70)
Muscular strength	1.71 (.30) **	4.23 (.20)	.52 (1.58–3.68)
Waist	2.21 (.24) **	3.61 (.31)	.42 (.52–2.24)
Energy level	1.50 (.17) **	4.23 (.20)	.66 (2.63–5.29)
Thighs	2.36 (.34) **	3.69 (.26)	.41 (.35–2.02)
Ears	3.29 (.27) *	4.15 (.22)	.47 (1.11–3.02)
Biceps	2.38 (.24) **	3.77 (.28)	.44 (.58–2.35)
Chin	3.07 (.22) **	4.08 (.21)	.42 (.42–2.10)
Body build	2.42 (.32) **	4.30 (.17)	.46 (.99–2.86)
Physical coordination	2.50 (.32) **	4.53 (.14)	.48 (1.19–3.13)
Buttocks	2.62 (.35) *	3.85 (.27)	.42 (.24–1.92)
Agility	1.85 (.29) **	4.38 (.21)	.52 (1.60–3.72)
Width of shoulders	2.93 (.32) *	4.08 (.26)	.41 (.23–1.87)
Arms	2.86 (.25)	3.54 (.29)	.39 (-.11–1.47)
Chest or breasts	2.50 (.27) **	3.77 (.23)	.42 (.50–2.21)
Appearance of eyes	3.00 (.31) *	3.92 (.23)	.40 (.08–1.70)
Cheeks /cheekbones	3.29 (.24)	3.84 (.25)	.39 (-.16–1.40)
Hips	2.29 (.29) **	3.77 (.20)	.44 (.73–2.51)
Legs	2.14 (.27) **	3.69 (.26)	.43 (.56–2.29)
Figure or physique	2.84 (.27) **	3.84 (.24)	.45 (.85–2.67)
Sex drive	2.42 (.37) **	3.92 (.21)	.42 (.47–2.18)
Feet	2.62 (.33)	3.54 (.31)	.40 (-.04–1.58)
Sex organs	2.84 (.27) *	3.84 (.24)	.41 (.22–1.89)
Appearance of stomach	2.14 (.31) *	3.31 (.29)	.41 (.23–1.88)
Health	1.42 (.13) **	4.53 (.18)	.81 (3.63–6.88)
Sex activities	2.14 (.43) **	3.76 (.28)	.41 (.37–2.04)
Body hair	2.50 (.34)	3.23 (.36)	.39 (-.22–1.34)
Physical condition	1.50 (.22) **	4.15 (.24)	.56 (1.89–4.15)
Face	3.00 (.26) **	4.08 (.18)	.42 (.46–2.16)
Weight	2.21 (.35) *	3.38 (.31)	.40 (.14–1.77)
General BES score	2.52 (.17) **	3.89 (.16)	.48 (1.23–3.18)

FMS patients assigned lower scores to all evaluated body features, relative to healthy controls. Differences were statistically significant across most items of the questionnaire, as well as in the general BES score (* p < .05, **p < .01 on the Mann-Whitney-Wilcoxon *U*-test). Data are presented as mean (SEM), and Cohen’s *d* (95% CI) is provided as a measure of effect size.

### Increased interoceptive awareness in FMS

We used the Body Perception Questionnaire (BPQ) to assess awareness of one’s own body processes, stress responses, autonomic nervous system reactivity and stress style. We found higher scores in the FMS sample relative to healthy controls in all addressed subscales, with large effect sizes and statistically significant differences (Student’s *t*-test for independent samples) on all of them with the sole exception of the stress style 1 subscale (Table 4; scores across all measured 94 items are provided in S1 Table). In addition, we found a strong and significant (p = .002) correlation (two-tailed Pearson’s correlation r = .75) between intensity of ongoing pain reported on the VAS and the awareness subscale of the BPQ in the FMS sample.

**Table 4:** Scores from FMS subjects and healthy controls on the Body Perception Questionnaire.

Domains	FMS	Healthy Controls	Effect size
Awareness	3.57 (.15) **	1.87 (.11)	.61 (2.28–4.73)
Stress response	3.76 (.11) **	1.78 (.11)	.87 (3.89–7.40)
*Autonomic Nervous System Reactivity*	2.59 (.17) **	1.35 (.07)	.51 (1.49–3.56)
Stress style 1	3.25 (.20)	2.55 (.38)	.40 (-.02–1.60)
Stress style 2	2.73 (.27) **	1.13 (.04)	.68 (2.76–5.49)

Scores from FMS patients were higher across all subscales, with large effect sizes and statistically different differences on most subscales (** p < .01 on the Student’s *t*-test for independent samples). Effect size is provided as Cohen’s *d* (95% confidence interval).

## Discussion

### Increased plasticity of the body schema

Internal representations of one’s own body include the notions of shape and contours of the own body, the location of the body parts and the boundaries between them [17,63–64]. Such representations are subject to substantial plasticity, as exemplified by the experience of perceiving a body part as alien and, conversely, by conditions of embodiment of external objects as one's own.

The RHI is a misperception in which tactile sensations are referred to an alien limb. The illusion is commonly employed for assessing plasticity of the body schema [45–49]. In the current study, the RHI could consistently be induced in participants from the FMS group, whose scores were significantly higher relative to healthy controls in questions addressing the perceptions of motor control over the fake hand and proprioceptive drift. Scores regarding the sense of ownership of the alien hand also were all higher in the FMS group, showing large effect sizes on the vast majority of items. Albeit still large, effect sizes were less pronounced on the *ownership* subscale, where between-group contrasts failed to attain statistical significance. Although the RHI directly addresses the easiness to embody a foreign object, the outcome of this procedure is best conceptualized as a measure of plasticity of embodiment of one’s own limb [46], as the illusion can also be induced successfully in conditions involving the converse, i.e. disembodiment of body parts, as found in somatoparaphenia [48,65] or in psychiatric patients involving dissociative states [56]. Previous studies have found direct correlation between the intensity of the RHI and plasticity of body representations measured with the Trinity Assessment of Body Plasticity in healthy subjects [66]. Interestingly, previous reports also have shown enhanced RHI in patients with low back pain [67–68], although its intensity may be rather variable in subjects suffering from CRPS [69]. On the other hand, studies conducted in CRPS patients have reported manifestations of disembodiment of the painful limb such as neglect-like symptoms [70–73] and a range of feelings of disassociation and lack of ownership [29]. These findings, including the present ones in people with FMS, raise the possibility that instability of the body schema might play a role in the pathophysiology of chronic pain.

The intensity of the RHI is sensitive to detection of visuotactile mismatch or inconsistencies between incoming sensory inputs from the actual hand and expected attributes based on stored somatorepresentations, e.g. regarding texture or position [45,74]. Studies show that in individuals suffering from eating disorders, who indeed experience the RHI more strongly than healthy controls [75–76], negative cognitive bias toward one’s body may compromise adequate updating of offline body representations by incoming sensory input [77–78]. An analogous mechanism may potentially operate in FMS, since such negative bias has been reported by others (cf below) and also found here in terms of low body esteem.

### Biased body representation in FMS

Disturbances in body perception are increasingly recognized as accompanying chronic pain states. Phantom limb pain, which genuinely exemplifies distorted representations of one’s own body, is commonly accompanied by the perception of the affected body part as altered in size or consistency, or placed in unusual or non-anatomic positions [28,79–81]. In addition, decreased tactile acuity and inability to delineate the body outline both have been reported to coincide with the distribution of pain in individuals suffering from chronic low back pain [82]. Alterations of body perception in CRPS patients include faulty estimation of the size of the affected limb [27], spatial mislocalization [22,30], or decreased tactile acuity with a direct correlation between body perception disturbance and pain [31]. Indeed, the progression of body image distortions in CRPS patients parallels that of pain [83–84], raising the question of whether the altered somatorepresentations may contribute to generating or maintaining chronic pain. Interestingly, this notion appears to receive support from the observation that therapeutic approaches based on rehabilitation of tactile acuity can reduce pain in patients suffering from CRPS or chronic low back pain [85–87].

Body image distortions may be driven not only by sensory inputs but also by body dissatisfaction. For example, somatosensory disturbances including impaired tactile acuity are commonplace in anorexia nervosa, a condition in which body dissatisfaction is pivotal [88–90]. In individuals with FMS, a previous report has shown alteration of the perceived image of the whole body using the 10-item Body Image Scale [35]. Here, we found that participants in the FMS group scored significantly lower than healthy controls on the 35-item Body Esteem Scale, showing low levels of satisfaction regarding a variety of diverse body parts and functional features. Lower scores were found in the FMS patient sample across the vast majority of body regions addressed by the survey, and statistically significant differences were found in scores assigned to 14 out of 18 body regions addressed by the BES. Furthermore, we found that the body site regarded by each patient as the primary focus of ongoing clinical pain was consistently perceived as less satisfactory in terms of BES scores than the rest of evaluated body parts.

Low body esteem is strongly characterized by body dissatisfaction [91–92], which arises from discrepancy between current perceived-self and ideal body model [93–94]. FMS is a form of chronic widespread pain and therefore not circumscribed to a particular body region. Lower scores on the BES indicate distorted, overall negative body perceptions, a finding that is in general agreement with prior quantitative and qualitative studies in people with this condition [95–96]. Further, pain severity and negative body perceptions are reportedly directly correlated [35]. This generalized form of distorted body perception is based on a biased cognitive appraisal of one’s own body. Our current study design did not permit us to address whether body image distortion was primary or secondary to chronic pain. However, there are indications that at least some conditioning factors such as childhood trauma or physical or emotional abuse, which may be causal to low self-esteem [97–98], precede widespread pain in FMS. Studies have reported that a history of childhood trauma or abuse may be commonly found in FMS patients relative to the general population [99–102] and even a risk factor of FMS [101]. Moreover, evidence suggests that childhood trauma is related with pain severity in FMS [101,103]. Interestingly, high rates of child abuse and stressful life events have been reported in a variety of forms of chronic pain, including generalized pain [104–106], pelvic pain and vulvodynia [107–108], chronic musculoskeletal pain [109], headache [110], and irritable bowel syndrome and gastro-intestinal conditions [111–113]. Whether or not preceded by stressful life events or other pathogenic route, our present results support the existence of a link between pain and negatively biased cognitions of one’s own body in FMS, including low body esteem.

### Increased interoceptive awareness

Pain is a salient experience that capitalizes attention and interferes with cognitive ad emotional processes [114]. Cognitive changes accompanying chronic pain often involve disproportionate attentional selection of pain and pain-associated information at the expense of the remaining sensory inputs [114–116]. Hypervigilance to pain is associated both with higher sensitivity to experimental pain [117–118] and greater clinical pain in FMS and other conditions [117,119]. Further, there is an emerging view that increased vigilance in people with FMS may not be circumscribed to painful inputs but rather represent a generalized, perceptual style of amplification of a variety of sensory information [120], including innocuous or auditory inputs [121]. In this perspective, in the current study we show that female patients with FMS exhibited increased interoceptive awareness relative to healthy individuals; supporting the notion that hypervigilance may not be limited to external sensory input but also involves an enhanced awareness of internal bodily cues. To our knowledge, only one prior recent study has addressed interoceptive awareness in a sample of FMS patients, by using The Multidimensional Assessment of Interoceptive Awareness questionnaire [122], which however failed to find significant differences with respect to their control sample. This apparent discordance may well be due to differences in sensitivity of the employed tools. In addition to higher scores on the BPQ, we found here that enhanced awareness of one’s own body processes as reflected by the scores on the *awareness* subscale was strongly associated with the intensity of ongoing clinical pain, a core clinical feature of FMS. Based on these findings, one may speculate that the coping style to pain in FMS patients involves perceptual amplification also including increased interoceptive awareness, or alternatively that perceptual amplification might lead to chronic pain in FMS.

### Is the ability to update negatively biased somatorepresentations impaired in FMS?

Body-referenced spatial representations, as a part of the spatial experience, derive from both on-line afferent somatosensory inputs subserved by short-term memory and off-line representations or embodied information stored as long-term memory [78,123–124]. Studies in eating disorders suggest that if the mutual influence between somatosensory inputs and off-line body representations is disrupted, then the contents of the stored reference frame fail to be adequately updated and the individual becomes locked to negative representations of their bodies, a model termed as *allocentric lock* [125–127]. A role in this disturbance is thought to be played by the connection between the amygdala and the hippocampal complex, due to the ability of the former to enhance long-term consolidation of emotionally arousing perceptions [128]. Here, we found evidence of negatively biased body image in persons suffering from FMS, which was accompanied by instability and increased plasticity of the body schema. Importantly, such biased cognition prevailed in FMS patients despite an ongoing state of sensory hypervigilance, including increased awareness to internal bodily cues. We suggest that disturbed embodiment as observed here in FMS patients may share fundamental analogies with the allocentric lock framework, where inadequate updating of stored, negatively biased off-line somatorepresentations may play be pivotal. Consistent with this view, brain imaging studies in FMS patients have found axonal metabolic dysfunction in the left hippocampus, including decreased myoinositol/creatine ratios and lower levels of choline and N-acetyl aspartate [129]. In addition, diffusion tensor imaging data from FMS patients have revealed increased fractional anisotropy indicating tissue complexity and neuronal disorganization increase in amygdalae and hippocampi among other loci [130], although it remains to be established whether such changes are primary or secondary to FMS.

### Limitations of the study

The asynchronous stroking condition in the RHI was omitted in the present study due to fatigue and difficulties maintaining mental focus throughout the procedure in the FMS population sample. However, this was unlikely to have a substantial impact on the interpretation of our findings, as these were based on comparisons between FMS subjects and age-matched, healthy individuals. On the other hand, primary data are presented as inter-group comparisons of individual items within questionnaires, rather than differences among multiple group means.

In summary, we present experimental evidence of disturbed embodiment in FMS that was characterized by instability of the body schema and negatively biased cognitions regarding one’s own body, in addition to increased vigilance to internal bodily cues. These pieces of evidence may be interpreted collectively in a framework centered upon the inability of incoming sensory inputs to adequately update negatively biased off-line somatorepresentations stored as long-term memory.

## Supporting information

S1 File Assessment of the RHI in fibromyalgia and healthy controls. (CSV)

S1 Table Individual data from FMS subjects and healthy controls on the Body Perception Questionnaire and all item data presented as mean (SEM). (DOCX)
