# Supplementary material for: Embodied pain in fibromyalgia: Disturbed somatorepresentations and increased plasticity of the body schema
Source: PLoS One. 2018 Apr 6;13(4):e0194534. doi: 10.1371/journal.pone.0194534 (PMC5889164; doi:10.1371/journal.pone.0194534)
Supplement: S1 Table — (DOCX) [file pone.0194534.s002.docx]

**Body Perception Questionnaire individual data.** These domains use 5-point Likert scale (1-never 5-always).

| **FMS subject** | 1 | 2 | 3 | 4 | 5 | 6 | 7 | 8 | 9 | 10 | 11 | 12 | 13 | 14 |
| --- | --- | --- | --- | --- | --- | --- | --- | --- | --- | --- | --- | --- | --- | --- |
| Awareness  domain | 2.89 | 2.71 | 3.89 | 3.22 | 3.20 | 4.67 | 3.96 | 3.80 | 4.11 | 3.80 | 4.02 | 3.44 | 2.71 | 3.60 |
| Stress Response  domain | 3.20 | 2.90 | 3.20 | 3.00 | 4.33 | 4.50 | 3.50 | 3.60 | 4.10 | 3.60 | 4.90 | 4.10 | 3.60 | 4.30 |
| ANSR  domain | 1.07 | 2.07 | 2.78 | 2.11 | 2.37 | 3.82 | 2.85 | 2.44 | 3.26 | 2.59 | 3.15 | 2.30 | 2.44 | 3.00 |
| Stress Style I  domain | 3.50 | 3.00 | 3.28 | 3.25 | 2.87 | 4.25 | 3.85 | 2.37 | 4.12 | 2.37 | 3.12 | 3.62 | 2.87 | 3.25 |
| Stress Style II  domain | 2.50 | 1.75 | 2.25 | 2.50 | 2.50 | 3.50 | 4.00 | 3.00 | 2.75 | 2.25 | 4.00 | 2.25 | 2.75 | 2.25 |

| **Control healthy subject** | 1 | 2 | 3 | 4 | 5 | 6 | 7 | 8 | 9 | 10 | 11 | 12 | 13 |
| --- | --- | --- | --- | --- | --- | --- | --- | --- | --- | --- | --- | --- | --- |
| Awareness  domain | 1.71 | 2.13 | 2.38 | 2.71 | 1.49 | 1.84 | 1.73 | 2.13 | 1.44 | 1.44 | 1.67 | 1.67 | 1.91 |
| Stress Response  domain | 1.40 | 2.60 | 2.80 | 2.20 | 1.20 | 1.70 | 1.70 | 1.60 | 1.00 | 2.30 | 1.50 | 1.80 | 1.10 |
| ANSR  domain | 1.00 | 1.44 | 1.04 | 1.89 | 1.30 | 1.19 | 1.33 | 1.67 | 1.22 | 1.26 | 1.52 | 1.41 | 1.22 |
| Stress Style I  domain | 2.62 | 2.62 | 2.87 | 2.87 | 3.00 | 2.62 | 2.00 | 2.75 | 2.62 | 2.62 | 1.75 | 2.50 | 2.37 |
| Stress Style II  domain | 1.00 | 1.00 | 2.00 | 1.00 | 1.00 | 1.00 | 1.00 | 1.00 | 1.25 | 1.00 | 1.00 | 1.50 | 1.00 |

|  | FMS | Healthy Controls | Effect size  Cohen´s *d* (95%CI) |
| --- | --- | --- | --- |
| *Awareness* | | | |
| 1.Swallowing frequently | 3.50 (0.37) | 2.84 (0.37) | 0.390  (-0.305, 1.257) |
| 2. A ringing in my ears | 3.28 (0.32)^++^ | 1.76 (0.28) | 0.427  (0.508, 2.217) |
| 3. An urge to cough to clear my throat | 3.71 (0.32)^++^ | 1.84 (0.15) | 0.471  (1.050, 2.935) |
| 4. My body swaying when I am standing | 3.85 (1.02)^++^ | 1.38 (0.18) | 0.549  (1.778, 3.975) |
| 5. My mouth being dry | 4.35 (0.22)^++^ | 2.15 (0.22) | 0.530  (1.623, 3.746) |
| 6. How fast I am breathing | 3.78 (0.23)^++^ | 2.15 (0.24) | 0.458  (0.906, 2.739) |
| 7. Watering or tearing of my eyes | 3.42 (0.35)^++^ | 1.84 (0.27) | 0.426  (0.490, 2.195) |
| 8. My skin itching | 3.92 (0.16)^++^ | 1.75 (0.25) | 0.563  (1.785, 4.047) |
| 9. Noises associated with my digestion e | 3.21 (0.35)^++^ | 1.84 (0.24) | 0.454  (0.867, 2.686) |
| 10. Eye fatigue or pain | 4.28 (0.16)^++^ | 2.07 (0.28) | 0.522  (1.549, 3.640) |
| 11. Muscle tension in my back and neck | 4.78 (0.11)^++^ | 2.46 (0.26) | 0.572  (1.966, 4.255) |
| 12. A swelling of my body or parts of my body | 3.57 (0.29)^++^ | 2.08 (0.28) | 0.440  (0.544, 2.305) |
| 13. An urge to urinate | 3.57 (0.32)^++^ | 2.15 (0.31) | 0.418  (0.361, 2.034) |
| 14. Tremor in my hands | 3.35 (0.30)^++^ | 1.53 (0.21) | 0.460  (0.933, 2.774) |
| 15. An urge to defecate | 3.35 (0.28)^++^ | 2.07 (0.27) | 0.421  (0.413, 2.098) |
| 16. Muscle tension in my arms and legs | 4.42 (0.17)^++^ | 1.84 (0.27) | 0.570  (1.953, 4.236) |
| 17. A bloated feeling because of water retention | 3.50 (0.37)^++^ | 1.84 (0.29) | 0.425  (0.477, 2.178) |
| 18. Muscle tension in my face | 3.64 (0.34)^++^ | 1.30 (0.17) | 0.497  (1.323, 3.314) |
| 19. Goose bumps | 2.71 (0.30)^+^ | 1.84 (0.24) | 0.402  (0.041, 1.650) |
| 20. Facial twitches | 2.85 (0.29)^++^ | 1.53 (0.18) | 0.433  (0.588, 2.321) |
| 21. Being exhausted | 4.85 (0.09)^++^ | 2.61 (0.26) | 0.570  (1.948, 4.229) |
| 22. Stomach and gut pains | 3.35 (0.24)^++^ | 1.76 (0.32) | 0.436  (0.634, 2.379) |
| 23. Rolling or fluttering my eyes | 2.45 (0.35)^++^ | 1.07 (0.07) | 0.429  (0.536, 2.253) |
| 24. Stomach distension or bloatedness | 3.78 (0.28)^++^ | 1.61 (0.21) | 0.501  (1.357, 3.362) |
| 25. Palms sweating | 2.35 (0.37) | 2.00 (0.32) | 0.387  (-0.495, 1.053) |
| 26. Sweat on my forehead | 3.21 (0.28)^+^ | 2.25 (0.32) | 0.411  (0.056, 1.703) |
| 27. Clumsiness or bumping into people | 3.57 (0.27)^++^ | 1.30 (0.17) | 0.530  (1.617, 3.738) |
| 28. Tremor in my lips | 2.35 (0.30)^++^ | 1.00 (0.00) | 0.447  (0.773, 2.562) |
| 29. Sweat in my armpits | 3.14 (0.29) | 2.38 (0.21) | 0.396  (-0.109, 1.475) |
| 30. Sensations of prickling, tingling, or numbness in my body | 4.00 (0.18)^++^ | 1.30 (0.17) | 0.679  (2.752, 5.469) |
| 31. The temperature of my face (especially my ears) | 3.14 (0.32)^++^ | 1.61 (0.21) | 0.441  (0.697, 2.462) |
| 32. Grinding my teeth | 3.21 (0.44)^+^ | 1.61 (0.21) | 0.419  (0.389, 2.068) |
| 33. General jitteriness | 4.30 (0.26)^++^ | 2.38 (0.21) | 0.499  (1.231, 3.230) |
| 34. Muscle pain | 4.85 (0.14)^++^ | 1.92 (0.23) | 0.676  (2.736, 5.443) |
| 35. Joint pain | 4.78 (0.11)^++^ | 1.84 (0.22) | 0.733  (3.120, 6.055) |
| 36. Fullness of my bladder | 3.14 (0.29) | 2.23 (0.36) | 0.398  (-0.040, 1.554 ) |
| 37. My eye movements | 2.61 (0.33)^+^ | 1.69 (0.36) | 0.405  (-0.075, 1.545) |
| 38. Back pain | 4.92 (0.07)^++^ | 2.53 (0.33) | 0.537  (1.678, 3.827) |
| 39. My nose itching | 3.28 (0.32)^+^ | 2.07 (0.32) | 0.408  (0.193, 1.828) |
| 40. The hair on the back of my neck "standing up" | 2.00 (0.29)^++^ | 1.00 (0.00) | 0.422  (0.430, 2.119) |
| 41. Needing to rest | 4.71 (0.12)^++^ | 2.61 (0.26) | 0.539  (1.699, 3.858) |
| 42. Difficulty in focusing | 4.50 (0.13)^++^ | 2.23 (0.28) | 0.543  (1.735, 3.910) |
| 43. An urge to swallow | 2.85 (0.40)^++^ | 1.30 (0.13) | 0.428  (0.524, 2.239) |
| 44. How hard my heart is beating | 3.57 (0.22)^++^ | 2.23 (0.20) | 0.449  (0.797, 2.594) |
| 45. Feeling constipated | 3.71 (0.38)^++^ | 1.69 (0.23) | 0.450  (0.809, 2.610) |
| *Stress Response* | | | |
| 46. Vascular responses such as my face becoming flushed or pallid, or feeling faint. | 3.71 (0.24)^++^ | 2.25 (0.27) | 0.448  (0.660, 2.455) |
| 47. Body posture shifts such as being hunched over, head down, and knees locked. | 3.76 (0.30)^++^ | 1.83 (0.20) | 0.489  (1.013, 2.972) |
| 48. Muscle tone or tremor such as arms and legs feeling weak, hands shaking, and lips quivering. | 4.07 (0.24)^++^ | 1.25 (0.13) | 0.669  (2.566, 5.243) |
| 49. Breathing more rapidly and shallowly, and having difficulty in catching my breath. | 3.64(0.19) ^++^ | 1.83 (0.24) | 0.505  (1.278, 3.299) |
| 50. Digestive responses including gastric distress, gas, cramps, and diarrhea. | 3.92 (0.22)^++^ | 1.75 (0.25) | 0.530  (1.508, 3.631) |
| 51. Difficulty in paying attention with my mind wondering or daydreaming. | 4.07 (0.19)^++^ | 2.25 (0.27) | 0.491  (1.144, 3.111) |
| 52. Difficulties in sensory abilities such as problems hearing, seeing, smelling, or feeling touch. | 3.14 (0.43)^++^ | 1.23 (0.12) | 0.443  (0.732, 2.507) |
| 53. Emotional problems such as more frequent feelings of depression, frustration, rage, or anger. | 4.35 (0.13)^++^ | 1.92 (0.26) | 0.581  (2.036, 4.361) |
| 54. Difficulty organizing my thoughts. | 3.64 (0.22)^++^ | 2.00 (0.27) | 0.454  (0.867, 2.686) |
| 55. Difficulty speaking clearly and understandably | 3.35 (0.38)^++^ | 1.53 (0.24) | 0.421  (0.420, 2.107) |
| *Autonomic Nervous System Reactivity* | | | |
| 56. I feel nauseous. | 2.57 (0.30)^++^ | 1.38 (0.18) | 0.421  (0.420, 2.107) |
| 57. I have difficulty coordinating breathing and eating. | 2.28 (0.24)^++^ | 1.07 (0.07) | 0.455  (0.88, 2.703) |
| 58. My nose is runny, even when I am not sick. | 2.78 (0.36) | 2.38 (0.38) | 0.387  (-0.483, 1.065) |
| 59. When I am eating, I have difficulty talking. | 2.85 (0.25)^+^ | 1.84 (0.37) | 0.402  (0.063, 1.674) |
| 60. My heart often beats irregularly. | 2.85 (0.25)^++^ | 1.61 (0.21) | 0.432  (0.574, 2.303) |
| 61. When I eat, food feels dry and sticks to my mouth and throat. | 2.57 (0.37)^++^ | 1.15 (0.10) | 0.428  (0.527, 2.242) |
| 62. I have "sour" stomach. | 2.85 (0.32)^+^ | 1.69 (0.26) | 0.411  (0.237, 1.882) |
| 63. I feel like vomiting. | 2.00 (0.20)^++^ | 1.15 (0.10) | 0.428  (0.521, 2.234) |
| 64. I feel shortness of breath. | 3.14 (0.25)^++^ | 1.25 (0.13) | 0.517  (1.509, 3.580) |
| 65. I have difficulty coordinating breathing with talking. | 2.71 (0.30)^++^ | 1.00 (0.00) | 0.482  (1.165, 3.094) |
| 66. When I eat, I have difficulty coordinating swallowing, chewing, and/or sucking with breathing. | 2.21 (0.26)^++^ | 1.00 (0.00) | 0.453  (0.854, 2.669) |
| 67. I have a persistent cough that interferes with my talking and eating. | 2.00 (0.36) | 1.46 (0.21) | 0.390  (-0.294, 1.268) |
| 68. I drool, especially when I am excited. | 2.21 (0.33) | 1.84 (0.22) | 0.388  (-0.426, 1.126) |
| 69. I gag from the saliva in my mouth. | 1.78 (0.26)^++^ | 1.00 (0.00) | 0.415  (0.308, 1.969) |
| 70. I produce a lot of saliva even when I am not eating. | 1.64 (0.24) | 1.38 (0.21) | 0.387  (-0.474, 1.074) |
| 71. I have difficulty adjusting my eyes to changes in illumination. | 4.07 (0.26)^++^ | 2.07 (0.28) | 0.468  (1.023, 2.898) |
| 72. I have chest pains. | 3.28 (0.19)^++^ | 1.15 (0.15) | 0.591  (2.117, 4.484) |
| 73. I gag when I eat. | 1.92 (0.22)^++^ | 1.00 (0.00) | 0.441  (0.702, 2.467) |
| 74. When I talk, I often feel I should cough or swallow the saliva in my mouth. | 2.78 (0.21)^++^ | 1.23 (0.16) | 0.487  (1.222, 3.173) |
| 75. I am constipated. | 3.78 (0.38)^++^ | 1.69 (0.26) | 0.451  (0.828, 2.633) |
| 76. I have indigestion. | 2.14 (0.23)^+^ | 1.46 (0.18) | 0.403  (0.077, 1.692) |
| 77. After eating I have digestive problems. | 2.92 (0.28)^++^ | 1.61(0.21) | 0.430  (0.548, 2.269) |
| 78. I have diarrhea. | 2.92 (0.37)^++^ | 1.38 (0.18) | 0.431  (0.565, 2.290) |
| 79. When I breathe, I feel like I cannot get enough oxygen. | 3.21 (0.33)^++^ | 1.38 (0.18) | 0.459  (0.916, 2.753) |
| 80. I have difficulty controlling my eyes. | 2.42 (0.29)^++^ | 1.30 (0.23) | 0.415  (0.314, 1.975) |
| 81. I get dizzy when urinating or having a bowel movement. | 1.57 (0.27)^+^ | 1.00 (0.00) | 0.400  (-0.006, 1.594) |
| 82. I have trouble focusing when I go into dimly or brightly illuminated places. | 3.71 (0.28)^++^ | 1.84 (0.24) | 0.463  (0.965, 2.818) |
| *Stress Style 1* | | | |
| 83. I approach the problem head-on. | 3.35 (0.22)^++^ | 4.38 (0.21) | 0.422  (-2.120, -0.431) |
| 84. I withdraw. | 2.38 (0.31)^+^ | 1.46 (0.24) | 0.412  (0.092, 1.741) |
| 85. I know that things will be better later, so I wait until I feel better before acting. | 3.00 (0.27) | 3.30 (0.32) | 0.442  (0.716, 2.487) |
| 86. I know that things will go better if I act immediately. | 2.64 (0.22) | 3.30 (0.30) | 0.395  (-1.466, 0.116) |
| 87. I feel mental tension. | 4.07 (0.26)^++^ | 2.92 (0.34) | 0.409  (0.193, 1.830) |
| 88. I feel frustrated. | 3.42 (0.35)^++^ | 2.07 (0.23) | 0.418  (0.361, 2.034) |
| 89. I feel insecure. | 3.92 (0.28)^++^ | 1.84 (0.19) | 0.497  (1.318, 3.307) |
| 90. I feel aimless. | 3.23 (0.37)^++^ | 1.15 (0.10) | 0.486  (1.103, 3.050) |
| *Stress Style 2* | | | |
| 91. I feel dizzy. | 3.07 (0.19)^++^ | 1.23 (0.16) | 0.537  (1.681, 3.832) |
| 92. I have difficulty speaking. | 3.21 (0.23)^++^ | 1.15 (0.15) | 0.539  (1.696, 3.853) |
| 93. I feel a tingling in my face. | 2.64 (0.26)^++^ | 1.00 (0.00) | 0.498  (1.325, 3.318) |
| 94. I feel my blood sugar drop. | 2.00 (0.37) | 1.15 (0.10) | 0.400  (0.016, 1.619) |

Differences were statistically significant in the vast majority of items (^+^ p<0.05, ^++^p<0.01 on the Mann-Whitney-Wilcoxon *U*-test). Data are presented as mean (SEM), and Cohen’s *d* (95% CI) is provided as a measure of effect size.
